# Using the POCT real-time PCR to detect *Clostridium difficile* in the environment to reduce the healthcare association infection

**DOI:** 10.1017/ash.2025.122

**Published:** 2025-09-03

**Authors:** FuChieh Chang, Chang-Pan Liu

**Affiliations:** 1 Infection Control Center, MacKay Memorial Hospital, Taipei city, Taiwan; 2 Division of Infectious Diseases, Department of Internal MedicineMacKay Memorial Hospital, Taipei city, Taiwan

## Abstract

**Introduction:** To avoid Clostridium difficile infection in the healthcare facility is an important work. There were many methods to do in the C. difficile infection (CDI) reduction bundle, including cleaning and disinfection. After cleaning and disinfection, we can do an environmental examination to check whether it contains C.difficile or not. Traditional, we did the culture to check but it have to wait 24-48 hours. This method was so slow, so in this study, we try to use the molecular methodology to detect C.difficile. **Methods:** We collected the specimen after 16 hours when the cleaning and disinfection. Then we used the POCT real-time PCR((POCKIT central C.difficile, GeneReach Biotechnology Corp, Taiwan)) and culture agar to detect whether C.difficile is present or not. In this study, we collected 48 specimens from CDI patients’ environments when they transferred to another space or left. **Results:** We found all the POCT real-time PCR results were the same compared to the culture results. That’s to say, the POCT real-time PCR can replace the culture method and improve the term around time on the diagnosis of C.diffiicle. **Conclusion:** The molecular method could replace the traditional culture due to it was quick and precise. Patients can’t wait for the culture result in clinical, especially in the ICU. Once delayed, the mortality rate would arise. In other words, the POCKIT central C.difficile is useful in clinical. It can be used to detect whether C.difficile survives on the surface or not. However, due to the limitation of the sample count, the statistical significance was not complete. So we will collect the sample to finish this study.

